# Supplementary material for: Targeted viral adaptation generates a simian-tropic hepatitis B virus that infects marmoset cells
Source: Nat Commun. 2023 Jun 16;14:3582. doi: 10.1038/s41467-023-39148-3 (PMC10276007; doi:10.1038/s41467-023-39148-3)
Supplement: Supplementary file 1 — Supplementary Information [file 41467_2023_39148_MOESM1_ESM.docx]

**Supplementary Information**

**for**

**Targeted viral adaptation generates a simian-tropic hepatitis B virus**

**that infects marmoset cells**

Yongzhen Liu^1^, Thomas R. Cafiero^1^, Debby Park^1^, Abhishek Biswas^1, 2^, Benjamin Y. Winer^1, 3^, Cheul H. Cho^4^, Yaron Bram^5^, Vasuretha Chandar^5^, Aoife K. O’Connell^6^, Hans P. Gertje^6^, Nicholas Crossland^6, 7^, Robert E. Schwartz^5^, Alexander Ploss^1, *^

^1^ Department of Molecular Biology, Princeton University, Princeton, NJ 08540, USA

^2^ Research Computing, Office of Information Technology, Princeton University, Princeton, NJ, USA

^3^ Memorial Sloan-Kettering Cancer Center, 1275 York Avenue, New York, NY 10065, USA

^4^ Visikol, Inc., Hampton, NJ 08827, USA

^5^ Division of Gastroenterology and Hepatology, Department of Medicine, Weill Cornell Medicine, New York, NY, USA.

^6^ National Emerging Infectious Diseases Laboratories, Boston University, Boston, MA, USA

^7^ Department of Pathology and Laboratory Medicine, Boston University Chobanian & Avedisian School of Medicine, Boston, MA, USA

* Correspondence should be addressed to A.P. ([aploss@princeton.edu](mailto:aploss@princeton.edu))

**Table of contents**

Supplementary tables 1-5......................................................................................................2-7 Supplementary figures 1-12...................................................................................................7-20

Supplementary references.....................................................................................................21

**Supplementary Table 1. NTCP sequences used for phylogenetic analyses in different species.**

| **Name** | **Scientific Species** | **Group** | **Family** | **NTCP protein ID from GenBank** |
| --- | --- | --- | --- | --- |
| *Human* | *Homo sapiens* | Hominoid | *Hominidae* | NP_003040.1 |
| *Sumatran orangutan* | *Pongo abelii* | Hominoid | *Hominidae* | XP_002824936.1 |
| Northern white-cheeked gibbon | *Nomascus leucogenys* | Hominoid | *Hominidae* | XP_030668498.1 |
| Pygmy chimpanzee | *Pan paniscus* | Hominoid | *Hominidae* | XP_003824149.1 |
| Chimpanzee | *Pan troglodytes* | Hominoid | *Hominidae* | XP_510035.2 |
| Western gorilla | *Gorilla gorilla* | Hominoid | *Hominidae* | XP_030857897.1 |
|  |  |  |  |  |
| Ugandan red colobus | *Piliocolobus tephrosceles* | Old World monkey | *Cercopithecidae* | XP_023038597.1 |
| Colobus | *Colobus angolensis palliatus* | Old World monkey | *Cercopithecidae* | XP_011805059.1 |
| Gray langur | *Semnopithecus sp* | Old World monkey | *Cercopithecidae* | ALX72759.1 |
| Black snub-nosed monkey | *Rhinopithecus bieti* | Old World monkey | *Cercopithecidae* | XP_017715073.1 |
| Golden snub-nosed monkey | *Rhinopithecus roxellana* | Old World monkey | *Cercopithecidae* | XP_010358049.1 |
| Hamadryas baboon | *Papio hamadryas* | Old World monkey | *Cercopithecidae* | ALX38774.1 |
| Olive baboon | *Papio anubis* | Old World monkey | *Cercopithecidae* | XP_003902029.1 |
| Drill | *Mandrillus leucophaeus* | Old World monkey | *Cercopithecidae* | XP_011856290.1 |
| Green monkey | *Chlorocebus sabaeus* | Old World monkey | *Cercopithecidae* | XP_007985313.1 |
| Grivet | *Chlorocebus aethiops* | Old World monkey | *Cercopithecidae* | ALX38772.1 |
| Sooty mangabey | *Cercocebus atys* | Old World monkey | *Cercopithecidae* | XP_011909339.1 |
| Rhesus monkey | *Macaca mulatta* | Old World monkey | *Cercopithecidae* | ALX38773.1 |
| Crab-eating macaque | *Macaca fascicularis* | Old World monkey | *Cercopithecidae* | AJT39805.1 |
| White-tufted-ear marmoset | *Callithrix jacchus* | New World monkey | *Cebidae* | XP_035116722.1 |
| Cotton-top tamarin | *Saguinus oedipus* | New World monkey | *Cebidae* | ALX38775.1 |
| Common squirrel monkey | *Saimiri sciureus* | New World monkey | *Cebidae* | ALX72399.1 |
| Panamanian white-faced capuchin | *Cebus imitator* | New World monkey | *Cebidae* | XP_017371957.2 |
| Ma's night monkey | *Aotus nancymaae* | New World monkey | *Cebidae* | XP_012293622.1 |
|  |  |  |  |  |
| Small-eared galago | *Otolemur garnettii* | Prosimian | *Galagidae* | XP_003797406.1 |
| Gray mouse lemur | *Microcebus murinus* | Prosimian | *Cheirogaleidae* | XP_012636625.1 |
| Coquerel's sifaka | *Propithecus coquereli* | Prosimian | *Indriidae* | XP_012511609.1 |
|  |  |  |  |  |
| Northern tree shrew | *Tupaia belangeri* |  | *Tupaiidae* | AFK93890.1 |
| Big brown bat | *Eptesicus fuscus* | Yangochiroptera | *Vespertillionidae* | XP_008137122.1 |
| Little brown bat | *Myotis lucifugus* | Yangochiroptera | *Vespertillionidae* | XP_006091092.1 |
| Brandt's bat | *Myotis brandtii* | Yangochiroptera | *Vespertillionidae* | XP_005878012.1 |
|  |  |  |  |  |
| Rabbit | *Oryctolagus cuniculus* |  | *Leporidae* | NP_001076237.1 |
| House mouse | *Mus musculus* |  | *Muridae* | NP_001348901.1 |
|  |  |  |  |  |
| European marmot | *Marmota marmota* |  | *Sciuridae* | XP_015346065.1 |
| Thirteen-lined ground squirrel | *Ictidomys tridecemlineatus* |  | *Sciuridae* | XP_005322900.1 |
| Naked mole-rat | *Heterocephalus glaber* | Ctenohystrica | *Bathyergidae* | XP_004837370.2 |
| Domestic guinea pig | *Cavia porcellus* | Ctenohystrica | *Caviidae* | XP_005008790.2 |
| Long-tailed chinchilla | *Chinchilla lanigera* | Ctenohystrica | *Chinchillidae* | XP_005399085.1 |
|  |  |  |  |  |

**Supplementary Table 2. Oligos for generation of NTCP orthologues and/or their humanized mutants for human, OWM, NWM and prosimian species.**

| **Primers** | **Oligo sequences (5’-3’)** |
| --- | --- |
| hNTCP-F | ATTTCCGGTGAATTCCTCGAGGCCGCCACCATGGGACTACAAGGACGATGACGATAAGatggaggcccacaacgc |
| hNTCP-R | CTCTTCGCCCTTAGACACCATCTCGCCCTTGCTCACggctgtgcaaggggagca |
| VSKGE-RFP-F | tgctccccttgcacagccGTGAGCAAGGGCGAGATGGTGTCTAAGGGCGAAGAG |
| VSKGE-RFP-R | CCAGACGCGTCTAATTAAGTTTGTGCCCCAGTTTG |
|  |  |
| CynoNTCP-F | ATTTCCGGTGAATTCCTCGAGGCCGCCACCATGGGACTACAAGGACGATGACGATAAGatggaggcccacaacgc |
| CynoNTCP-R | CACGGTGCCCTCCATGTAcAGCTTCATGTGCATGTTCTCCTTAATCAGCTCTTCGCCCTTAGACACCATCTCGCCCTTGCTCACggctgtgcaaggggagc |
| CynoNTCP^R158G^-F | GACAAGGTGCCCTATGGAgGCATCATATTATCACTGGTCCCG |
| CynoNTCP^R158G^-R | CGGGACCAGTGATAATATGATGCcTCCATAGGGCACCTTGTC |
| CynoNTCP^G157K,R158G^-F | CCTGAAGGACAAGGTGCCCTATAAAGGCATCATATTATCACTGGTCCC |
| CynoNTCP^G157K,R158G^-R | GGGACCAGTGATAATATGATGCCTTTATAGGGCACCTTGTCCTTCAGG |
| CynoNTCP^R158G,L161I^-F | GAAGGACAAGGTGCCCTATGGAGGCATCATAATATCACTGGTCCCGGTTCTC |
| CynoNTCP^R158G,L161I^-R | GAGAACCGGGACCAGTGATATTATGATGCCTCCATAGGGCACCTTGTCCTTC |
| CynoNTCP^R158G,I160V^-F | GAAGGACAAGGTGCCCTATGGAGGCATCGTGTTATCACTGGTCCCGGTTCTC |
| CynoNTCP^R158G,I160V^-R | GAGAACCGGGACCAGTGATAACACGATGCCTCCATAGGGCACCTTGTCCTTC |
| CynoNTCP^R158G,P165L^-F | GAAGGACAAGGTGCCCTATGGAGGCATCATATTATCACTGGTCCTGGTTCTCATTCCTTGCACCATAG |
| CynoNTCP^R158G,P165L^-R | CTATGGTGCAAGGAATGAGAACCAGGACCAGTGATAATATGATGCCTCCATAGGGCACCTTGTCCTTC |
| CynoNTCP^h157-165^-F | GAAGGACAAGGTGCCCTATAAAGGCATCGTGATATCACTGGTCCTGGTTCTCATTCCTTGCACCATAGG |
| CynoNTCP^h157-165^-R | CCTATGGTGCAAGGAATGAGAACCAGGACCAGTGATATCACGATGCCTTTATAGGGCACCTTGTCCTTC |
| CynoNTCP^Q84R, N86K^-F | CTTTGTGCTGGGCAAGGTCTTCCGGCTGAAGAACATTGAGGCGCTGGCCAT |
| CynoNTCP^Q84R, N86K^-R | ATGGCCAGCGCCTCAATGTTCTTCAGCCGGAAGACCTTGCCCAGCACAAAG |
|  |  |
| SqMNTCP-F | ATTTCCGGTGAATTCCTCGAGGCCGCCACCATGGGACTACAAGGACGATGACGATAAGatggatgcccacaatgtgt |
| SqMNTCP-R | CACGGTGCCCTCCATGTAcAGCTTCATGTGCATGTTCTCCTTAATCAGCTCTTCGCCCTTAGACACCATCTCGCCCTTGCTCACggctttgcaaggggagcac |
| SqMNTCP^G158R^-F | GACAAGGTGCCCTATGGACGCATCATGATATCACTGATCCT |
| SqMNTCP^G158R^-R | AGGATCAGTGATATCATGATGCGTCCATAGGGCACCTTGTC |
| SqMNTCP^Q84R^-F | TGGGCAAGGTCTTCCgGCTGAACAAAATTGAGGCAC |
| SqMNTCP^Q84R^-R | GTGCCTCAATTTTGTTCAGCcGGAAGACCTTGCCCA |
| SqMNTCP^K87N^-F | CAAGGTCTTCCAGCTGAACAAcATTGAGGCACTGGCCATC |
| SqMNTCP^K87N^-R | GATGGCCAGTGCCTCAATgTTGTTCAGCTGGAAGACCTTG |
| SqMNTCP^N86K,K87N^-F | GCAAGGTCTTCCAGCTGAAaAAcATTGAGGCACTGGCCAT |
| SqMNTCP^N86K,K87N^-R | ATGGCCAGTGCCTCAATgTTtTTCAGCTGGAAGACCTTGC |
| SqMNTCP^Q84R,N86K,K87N^-F | GCTGGGCAAGGTCTTCCgGCTGAAaAAcATTGAGGCACTGGCCA |
| SqMNTCP^Q84R,N86K,K87N^-R | TGGCCAGTGCCTCAATgTTtTTCAGCcGGAAGACCTTGCCCAGC |
|  |  |
| marmosetNTCP-F | ATTTCCGGTGAATTCCTCGAGGCCGCCACCATGGGACTACAAGGACGATGACGATAAGatggaggcccacaacgtg |
| marmosetNTCP-R | CACGGTGCCCTCCATGTAcAGCTTCATGTGCATGTTCTCCTTAATCAGCTCTTCGCCCTTAGACACCATCTCGCCCTTGCTCACgaaatgctggagagactcagg |
|  |  |
| prosimianNTCP-F | ATTTCCGGTGAATTCCTCGAGGCCGCCACCATGGGACTACAAGGACGATGACGATAAGatggaggcccacaatgc |
| prosimianNTCP-R | CACGGTGCCCTCCATGTAcAGCTTCATGTGCATGTTCTCCTTAATCAGCTCTTCGCCCTTAGACACCATCTCGCCCTTGCTCACgagctcctggagttgttcttc |

**Supplementary Table 3. Oligos for generation of HBV/WMHBV chimeric virus clones.**

| **Primers** | **Oligo sequences (5’-3’)** |
| --- | --- |
| HBV-gtD-WMHBV preS1[1-48]-direct mutation-F | ttgggaacaagatctacagcatgggactcaaccagtcaacgttcaacccgctgggattctttcccagtcatcagctggaccccctgttcaaggccaatgcaggctcagcagattgggacaaaaatccaaacaaggacccttggccacaagcccacgacacggcagctggagcattcgggctgg |
| HBV-gtD-WMHBV preS1[1-48]-direct mutation-R | ccagcccgaatgctccagctgccgtgtcgtgggcttgtggccaagggtccttgtttggatttttgtcccaatctgctgagcctgcattggccttgaacagggggtccagctgatgactgggaaagaatcccagcgggttgaacgttgactggttgagtcccatgctgtagatcttgttcccaa |
|  |  |

**Supplementary Table 4. Oligos for generation of pLVX^ΔCMV^-1.3-mer-HBV.**

| **Primers** | **Oligo sequences (5’-3’)** |
| --- | --- |
| pLVX-CMV-IRES-Puro-CMV-KO-F | acagggacagcagagatccagtttatcgatggatcccgcccctctccctcccccccccctaacgttactggccgaag |
| pLVX-CMV-IRES-Puro-CMV-KO-R | cttcggccagtaacgttaggggggggggagggagaggggcgggatccatcgataaactggatctctgctgtccctgt |
|  |  |
| CalI-HBV-1.3-mer-F | agggacagcagagatccagtttatTATTAACAGGCCTATTGATTGGAAAGT |
|  |  |
| HBV-1.3-mer-BamHI-R | gagggagaggggcgCAATGCTCAGGAGACTCTAAG |
|  |  |

**Supplementary Table 5.** Probe, antibodies, and antigen retrieval conditions for chromogenic ISH-IHC combination assay performed in this study.

| **Seq. Order** | **Ag Target** | **Species Origin** | **Clone** | **Manu-facturer** | **Cata-log/lot #** | **Primary probe or antibody Dilution** | **Antigen Retrieval (Ventana)** | **Incu-bation Temp.** | **Incuba-tion Period** | **Chromo- gen** |
| --- | --- | --- | --- | --- | --- | --- | --- | --- | --- | --- |
| Three-plex | | | | | | | | | | |
| 1 | HBV RNA | N/A | N/A | Molecular instruments | LSA204 | Pre-dilute | CC1 | 42°C | 2 Hrs. | DAB |
| 2 | HBV Core antigen | N/A | N/A | Lifespan Biosciences | LS-c312204 | 1:100 with amplification | CC1 | RT | 2 Hrs. | Purple |
| 3 | RFP | Rb | Polyclonal | Thermofisher Scientific | R10367 | 1:150 | CC1 | 37 | 1 Hr. | Yellow |
| Positive control Probe | UBC RNA | *Mus musculus | N/A | Molecular instruments | N451GP | Pre-dilute | CC1 | 42°C | 2 Hrs. | DAB |
| Negative control probe | dapB RNA | Bacillus subtilis | N/A | Molecular instruments | H307GP | Pre-dilute | CC1 | 42°C | 2 Hrs. | DAB |
| CC1-Cell conditioning 1-Tris based antigen retrieval buffer; HBV-Hepatitis B Virus; RFP-red fluorescent protein; N/A-not applicable; *Highly conserved sequence cross reacts with human and marmoset UBC gene. | | | | | | | | | | |


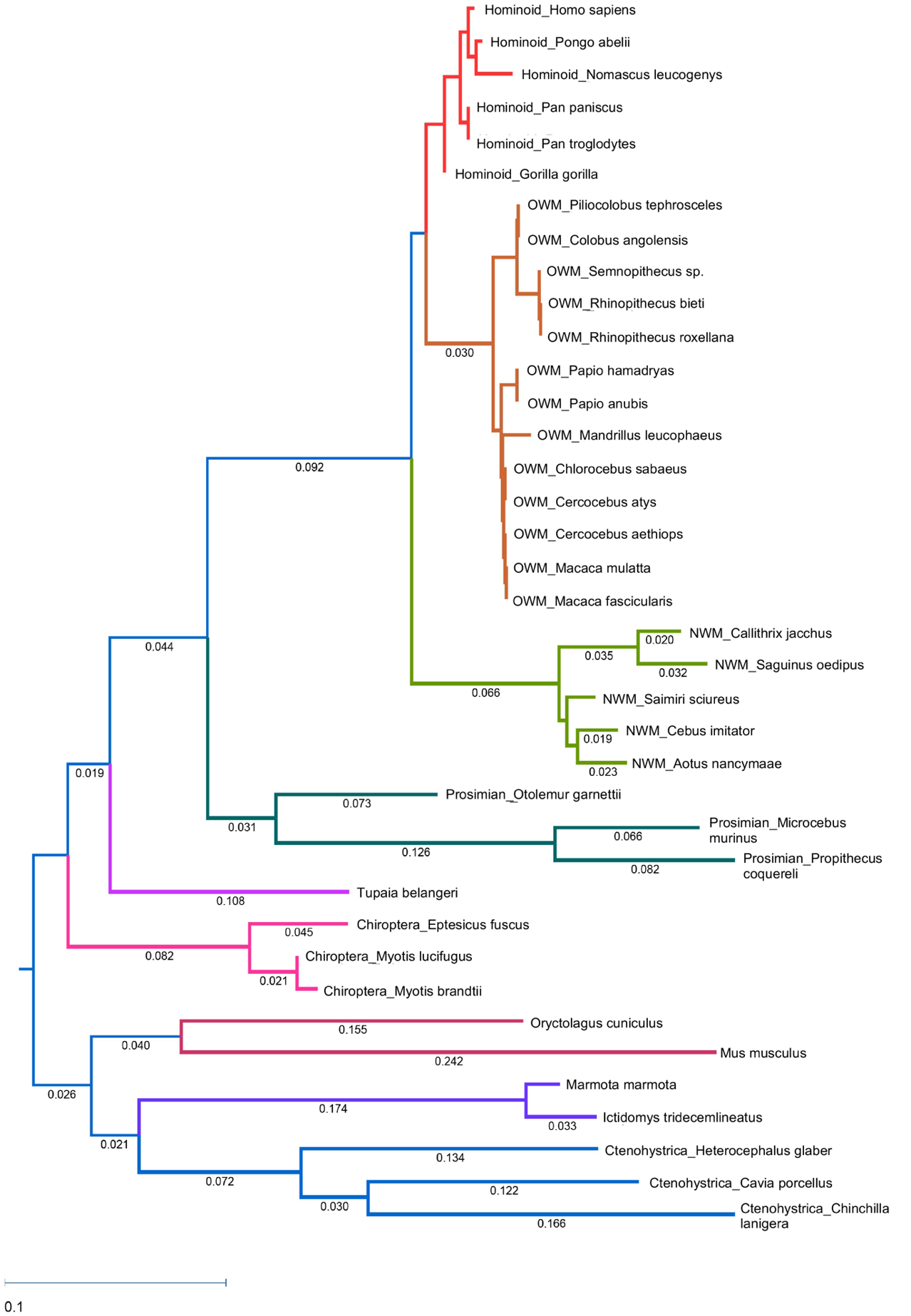


**Supplementary Figure 1.** Phylogenetic analyses of NTCP protein sequences from diverse mammalian species. The phylogenies were built using a RAXML alignment of orthologous amino acid sequences of NTCPs from 38 species. OWM, Old World monkey; NWM, New World monkey.


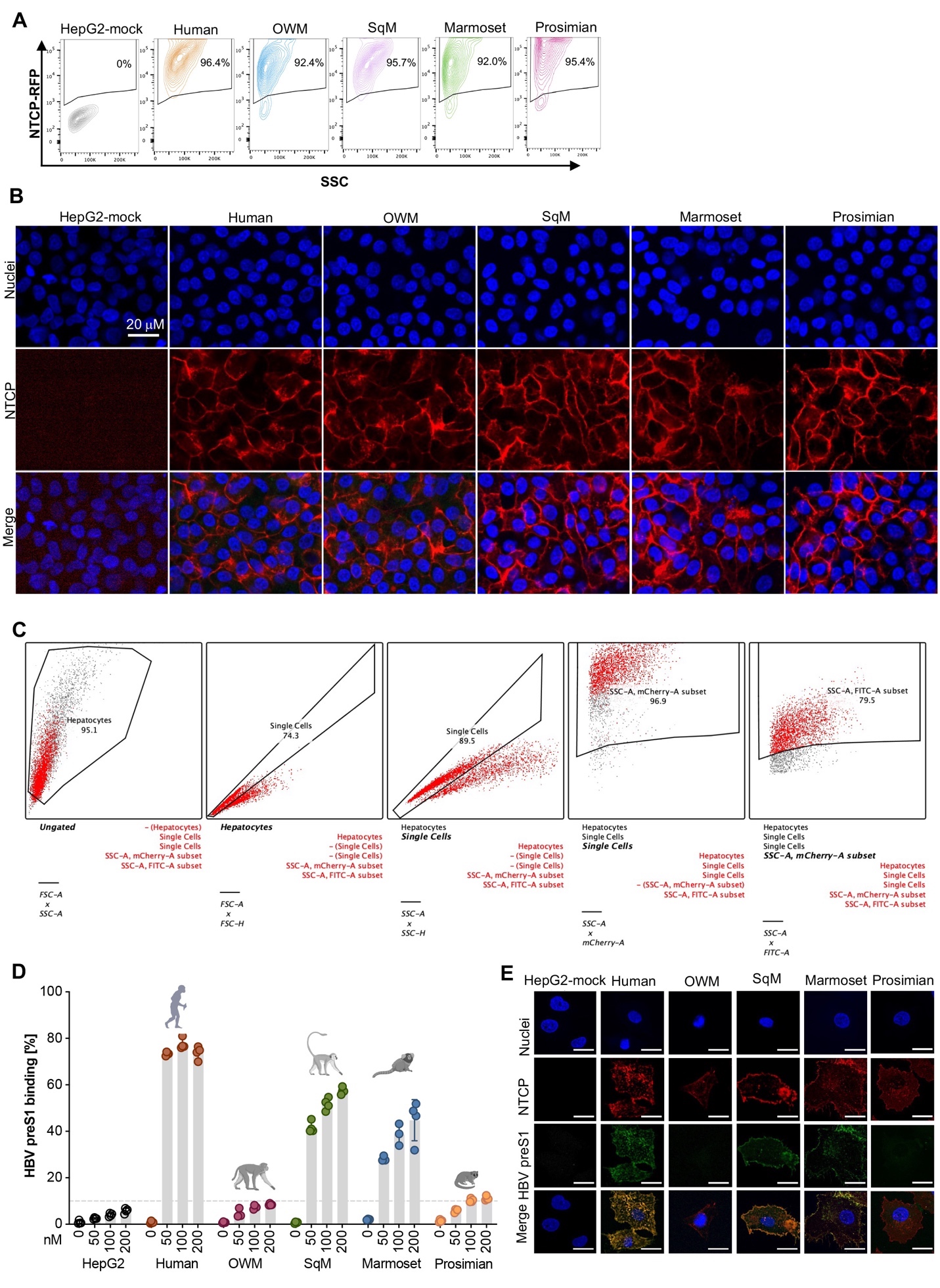


**Supplementary Figure 2.** (**A**) Representative flow cytometry data of NTCP expression in HepG2 cells. HepG2 cells were transduced with NTCP orthologues of the indicated species fused C-terminally to RFP. The frequency of NTCP-expressing cells was determined by quantifying RFP+ cells following lentiviral transduction. The data was analyzed by FlowJo (version 10) and this experiment was repeated independently four times. (**B**) Representative confocal imaging showing the ectopic NTCP expression of different species. The experiments were repeated twice independently with similar results and the representative images are shown. (**C**) Flow cytometric gating strategy for HBV/WMHBV preS1 binding assays. Binding efficiencies are expressed as the frequency of FITC+ cells within the NTCP-RFP population. (**D**) HepG2 cells transduced with NTCP orthologues of the indicated species were incubated with the HBV preS1 peptide (AAs 1-48) labeled with FITC at different concentrations (0, 50, 100 and 200 nM). Binding efficiencies are expressed as the frequency of FITC+ cells within the NTCP-RFP population. The binding percentage for mock HepG2 cells are shown as the baseline for this assay. Primate icons were created with BioRender.com. Gray bars depict mean of 4 biologically independent experiments and error bars represent s.e.m. The dash line indicates the lower limit of the assay based on the control groups. (**E**) Representative confocal imaging showing the binding of HBV preS1 peptide (200 nM). The scale bar is 20 μm. The experiments were repeated twice independently with similar results and the representative images were shown. HepG2 cells transduced with NTCP orthologues of the indicated species were incubated with the HBV preS1 peptide (AAs 1-48) labeled with FITC (200 nM) for imaging analysis by confocal microscopy (Nikon A1R-STED).


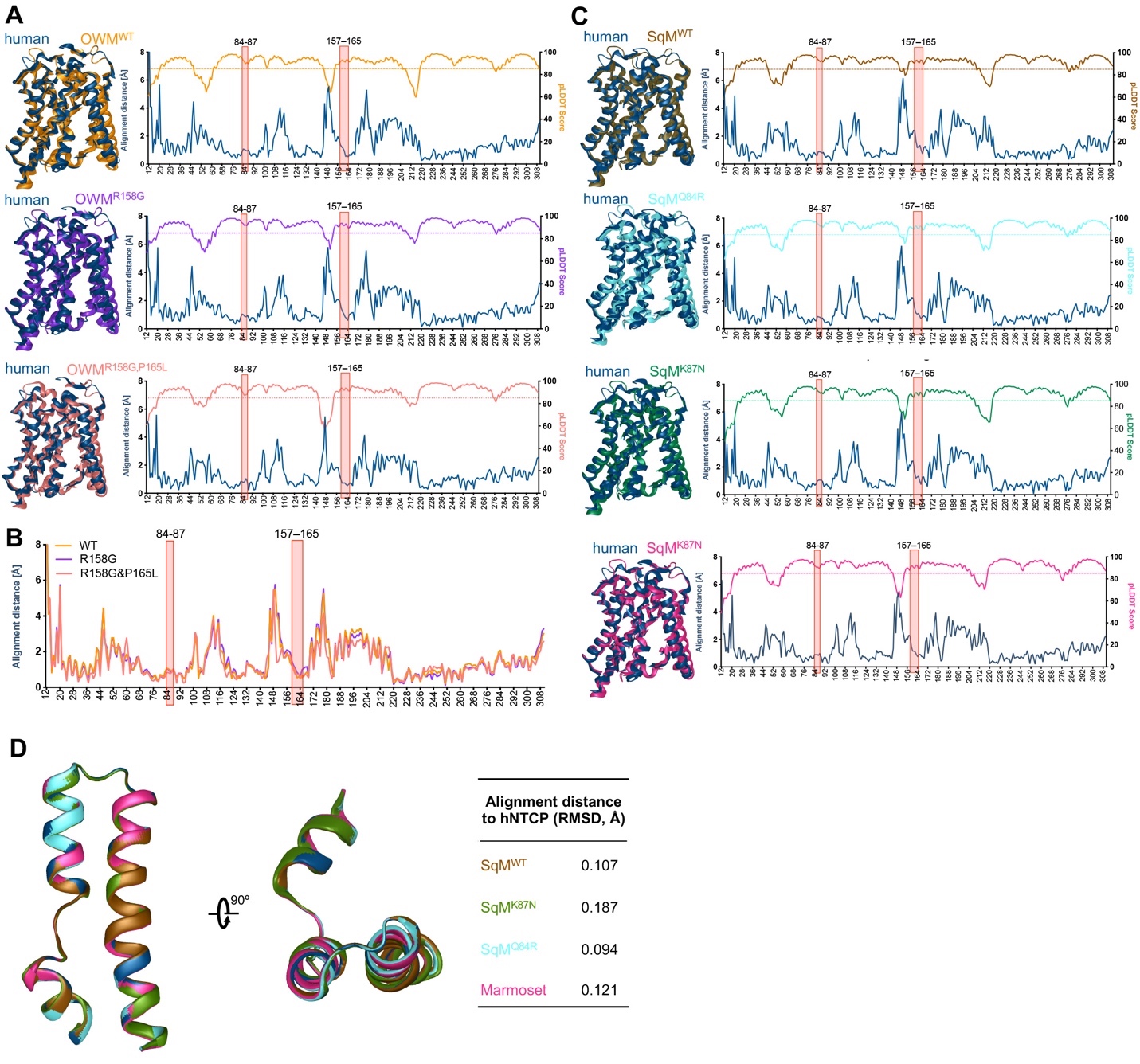


**Supplementary Figure 3. (A)** AlphaFold predictions^1^ of OWM^WT^, OWM^R158G^, OWM^R158G, P165L^ and marmoset NTCP structures and their similarity to the cryo-EM hNTCP structure (PDB: 7PQG). **(B)** Alignment distance of hNTCP compared among OWM^WT^, OWM^R158G^ and OWM^R158G, P165L^. Boxed in red are the regions that are critical for HBV entry. pLDDT score is used to indicate the confidence levels produced by AlphaFold^1^. **(C)** AlphaFold predictions^1^ of SqM^WT^, SqM^Q84R^, SqM^K87N^ and marmoset NTCP structures and their similarity to the cryo-EM hNTCP structure (PDB: 7PQG). Boxed in red are the regions critical for HBV entry. The pLDDT score is used to indicate the confidence levels produced by AlphaFold. (**D**) Structure alignment for the aa84-87 region and the surrounding transmembrane 2 and transmembrane 3 of SqM^WT^, SqM^Q84R^, SqM^K87N^ and marmoset NTCP. RMSD, similarity assay conducted by root mean squared deviation.


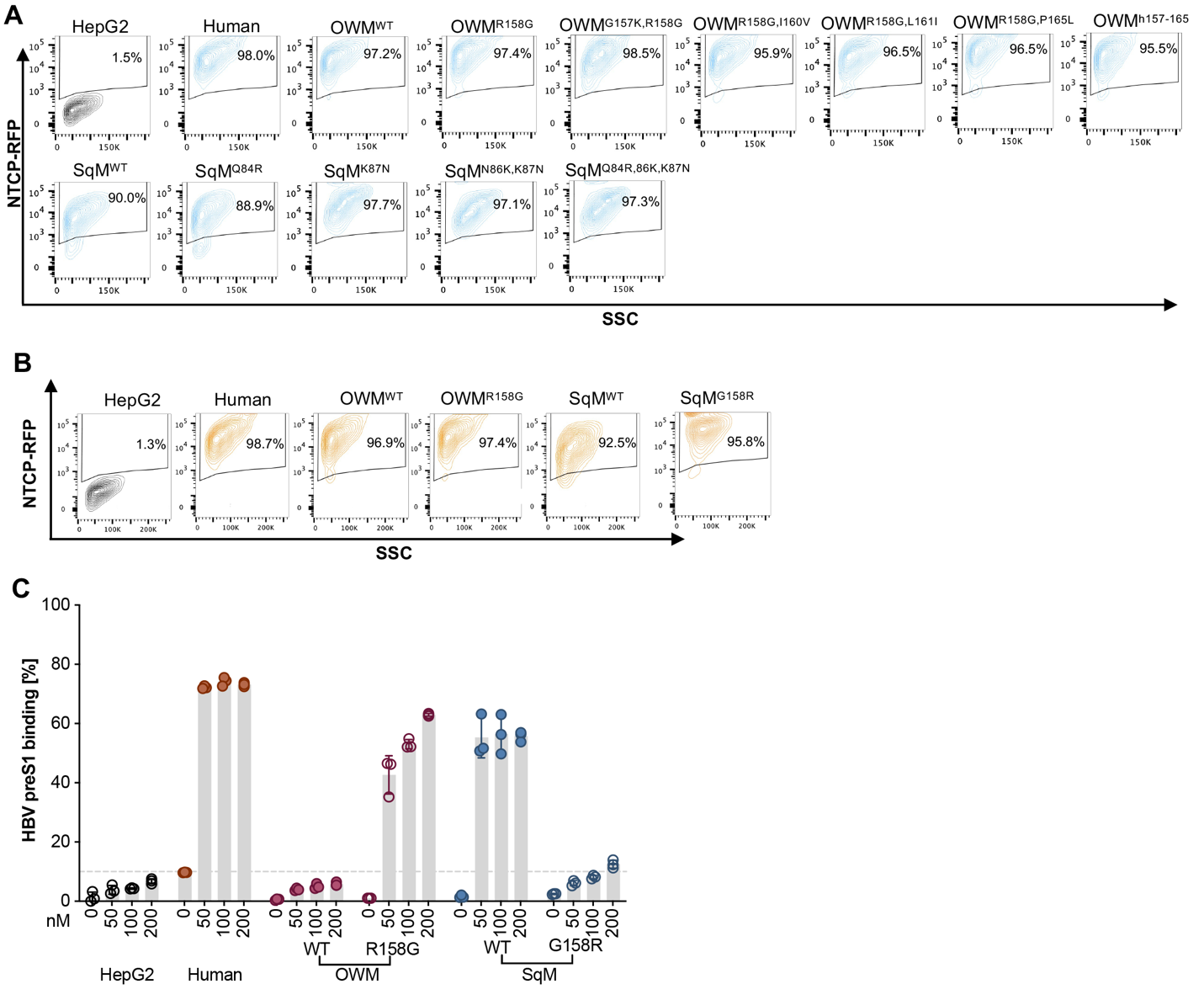


**Supplementary Figure 4.** (**A**) Quantification of (mutant) NTCP expression by flow cytometry. HepG2 cells were transduced with lentivirus expressing NTCP orthologues of the indicated species fused C-terminally with RFP. The frequency of NTCP-expressing cells was determined by quantifying RFP+ cells following lentiviral transduction. (**B**) Quantification of (mutant) NTCP expression for OWM R158G and NWM G158R by flow cytometry. HepG2 mock and hNTCP act as the negative and positive control, respectively. (**C**) G158 is necessary for HBV binding. HepG2 cells transduced with (mutant) NTCP orthologues were incubated with the HBV preS1 peptide (AAs 1-48) labeled with FITC at different concentrations (0, 50, 100 and 200 nM). Binding efficiencies are expressed as the frequency of FITC+ cells within the NTCP-RFP population. The data were analyzed by FlowJo (version 10). The dash line indicates the lower limit of the assay based on the control groups. Gray bars depict mean of 3 biologically independent experiments and error bars represent s.e.m.


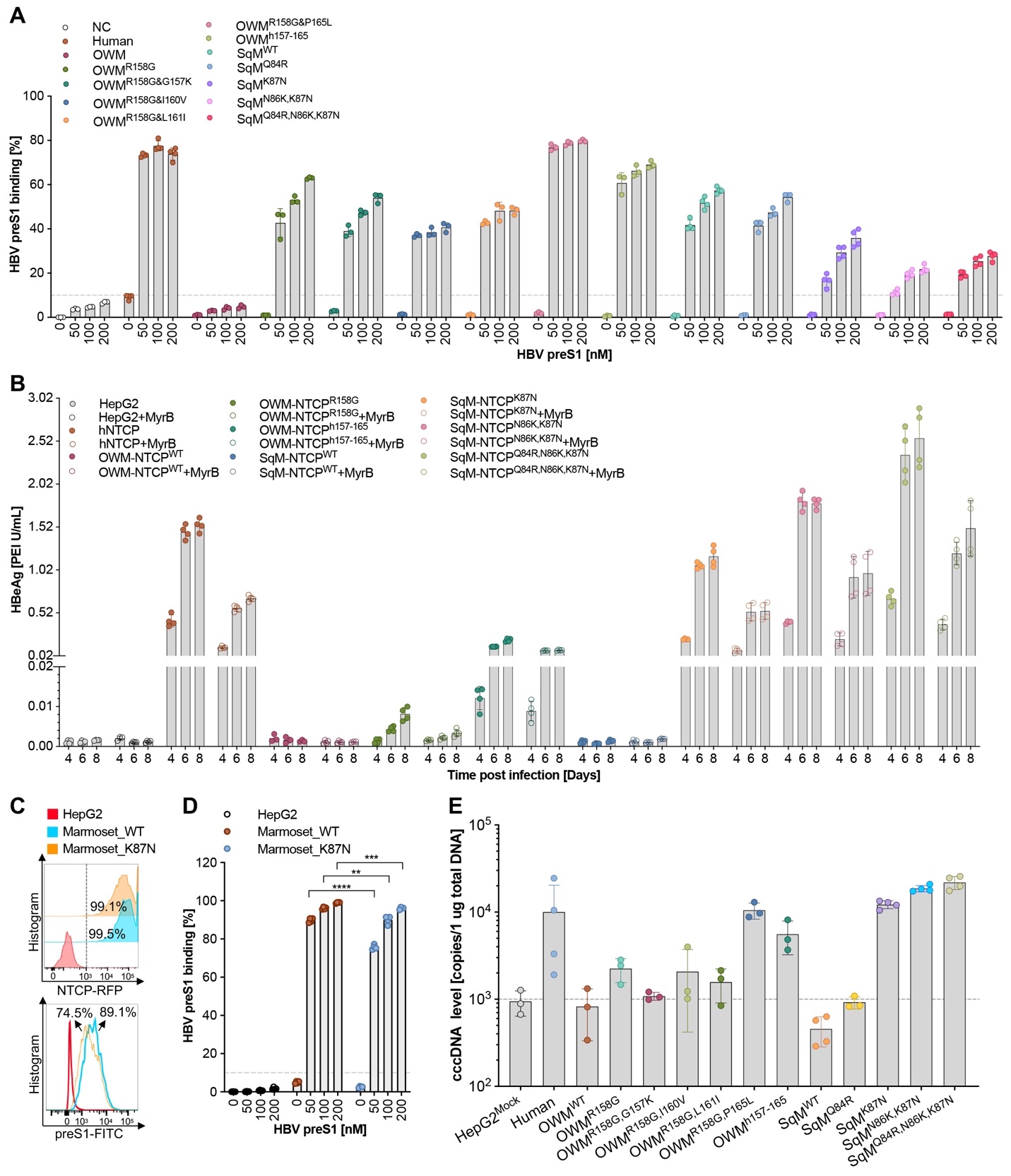


**Supplementary Figure 5.** (**A**) HBV binding to NTCP orthologues from human, OWM, NWM, and their variants. HepG2 cells transduced with (mutant) NTCP orthologues of the indicated species were incubated with the HBV preS1 peptide (AAs 1-48) labeled with FITC under different concentrations (0, 50, 100 and 200 nM). Binding efficiencies are expressed as the frequency of FITC+ cells within the NTCP-RFP population. The dash line indicates the lower limit of the assay based on the control groups. The data was analyzed by FlowJo (version 10). Gray bars depict mean of 3 biologically independent experiments (n=4 for human, SqM^WT^, SqM^K87N^, SqM^N86K,K87N^ and SqM^Q84R,N86K,K87N^ groups) and error bars represent s.e.m. The binding percentage for mock HepG2 cells are shown as the baseline for this assay. (**B**) HBV infections (MOI = 6000) were analyzed in HepG2 cells expressing NTCP orthologues from human, OWM, NWM, and their variants in the presence or absence of the competitive entry inhibitor Myrcludex B. Limit of detection = ca 0.05 PEI units/mL. Gray bars depict the mean of 4 biologically independent experiments (n=3 for OWM-NTCP^WT^ group) and error bars represent s.e.m. (**C&D**) HBV binding to NTCP orthologues from marmoset and its variant K87N. HepG2 cells transduced with two NTCP orthologues were incubated with the 5’ myristoylated and 3’ FITC-labeled preS1 peptides (AAs 2-48, 200 nM). Binding efficiencies are expressed as the frequency of FITC+ cells within the NTCP-RFP population. The data was analyzed by FlowJo (version 10). The binding percentage for mock HepG2 cells are shown as the baseline (dash line) for this assay. Gray bars depict mean of 4 independent experiments and error bars represent s.e.m. Unpaired t test, two-tailed. ** p = 0.0013; *** p = 0.0001;. **** p < 0.0001. (**E**) Intracellular cccDNA levels were determined at the endpoint (day 8) by RT-qPCR. The dash line indicates the lower limit of the assay based on the control group. The gray bars represent the mean of 3 biologically independent experiments (n= 4 for human, SqM^WT^, SqM^K87N^, SqM^N86K,K87N^ and SqM^Q84R,N86K,K87N^ groups) and error bars represent s.e.m.


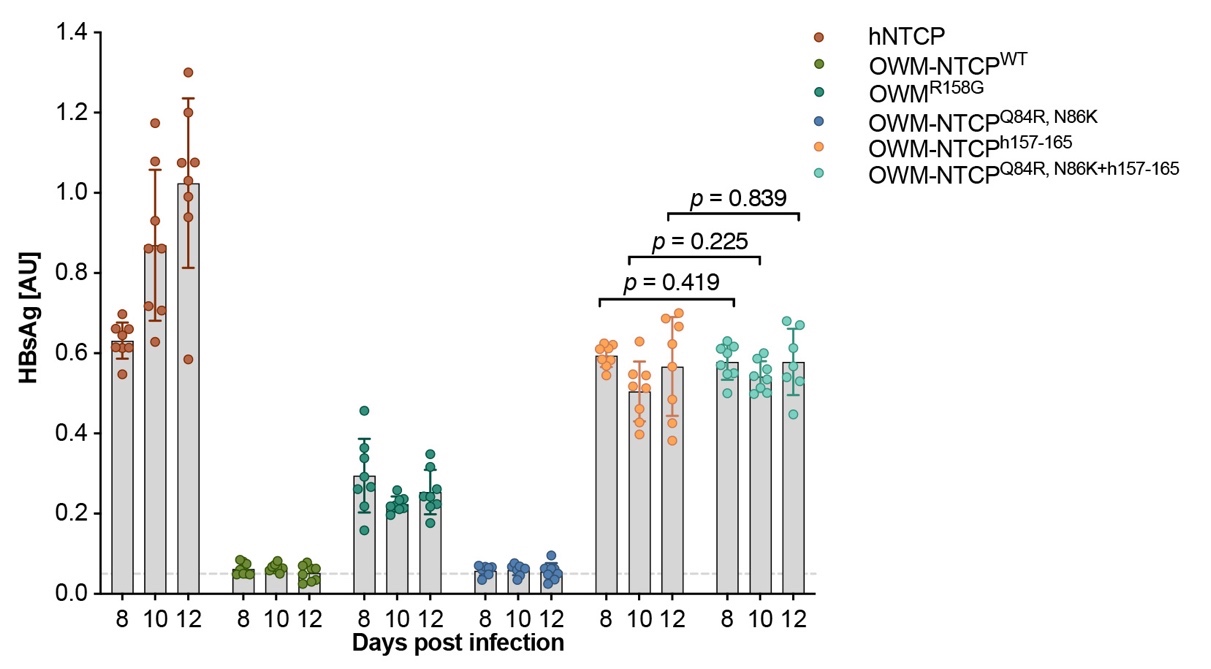


**Supplementary Figure 6.** HBsAg detection in supernatants of HBV-infected (MOI = 6000) HepG2 cells expressing different NTCP orthologues. The dash line shows the lower limit of the detection. OWM, old world monkey; WT, wild type. Gray bars depict mean of 8 independent experiments and error bars represent s.e.m. Unpaired t test, two-tailed.


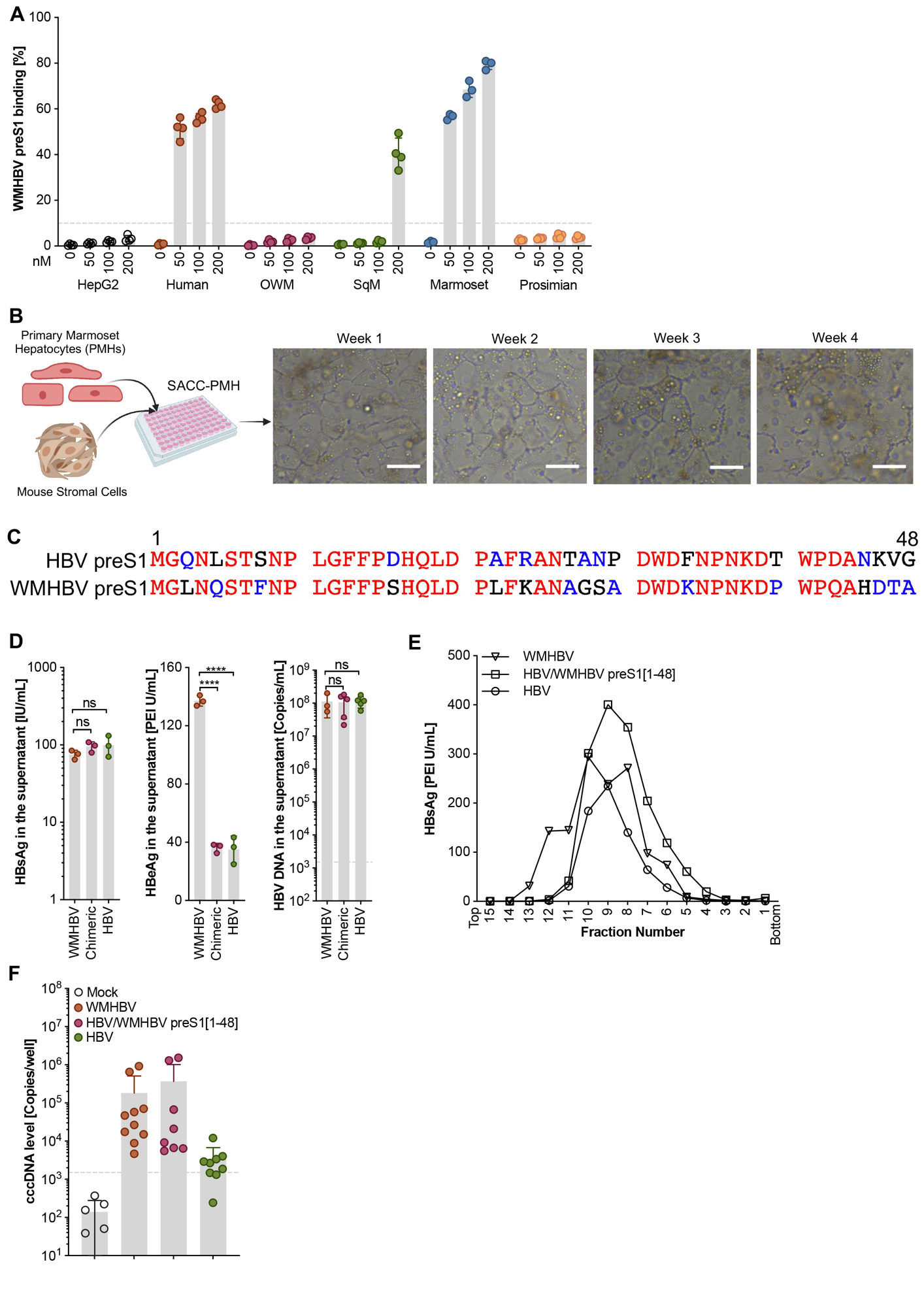


**Supplementary Figure 7.** (**A**) Binding of WMHBV preS1 to NTCP orthologues from human, OWM, NWM, and their variants. HepG2 cells transduced with (mutant) NTCP orthologues of the indicated species were incubated with the HBV preS1 peptide (AAs 1-48) labeled with FITC at different concentrations (0, 50, 100 and 200 nM). Binding efficiencies are expressed as the frequency of FITC+ cells within the NTCP-RFP population. The data was analyzed by FlowJo (version 10). Gray bars depict the mean of 4 biologically independent experiments (n=3 for marmoset group) and error bars represent s.e.m. The dash line indicates the lower limit of the assay based on the control groups. (**B**) Schematic (Created with BioRender.com.) of the self-assembling primary marmoset hepatocyte (SACC-PMH) co-culture system for HBV/WMHBV replication or infection. SACCs were established by plating PMHs with 3T3-J2 cells. Representative bright field images were taken 1, 2, 3 and 4 weeks after cell seeding. The scale bar is 50 µm. (**C**) Amino acid sequence alignment of residues 1-48 of HBV and WMHBV preS1. (**D**) HBsAg, HBeAg and HBV DNA levels in the supernatant as detected by ELISA and qPCR after transfection with WMHBV, HBV or HBV/WMHBV preS1[1-48] (“chimeric”) infectious clones into HepG2 cells. Gray bars depict the mean of 3 biologically independent experiments (n=5 for HBV DNA detection in chimeric and HBV groups) and error bars represent s.e.m. Unpaired t test, two-tailed. **** p < 0.0001. The dash line indicates the lower limit of the assay based on the control groups. (**E**) Biochemical characterization of WMHBV, HBV or HBV/WMHBVpreS1[1-48] chimeric viruses by sucrose density gradient centrifugation followed by HBsAg ELISA. The gradient was split into 1.3 mL fractions from the bottom to the top. (**F**) Intracellular cccDNA levels were determined at the endpoint (day 21) to evaluate virion infectivity in primary marmoset hepatocytes (PMHs). Uninfected PMHs were used as a control. Gray bars depict means of biologically independent experiments (n=6, 10, 8, 9 for mock, WMHBV, HBV/WMHBV preS1[1-48] and HBV, respectively) and error bars represent s.e.m. The dash lines show the lower limit of the detection.


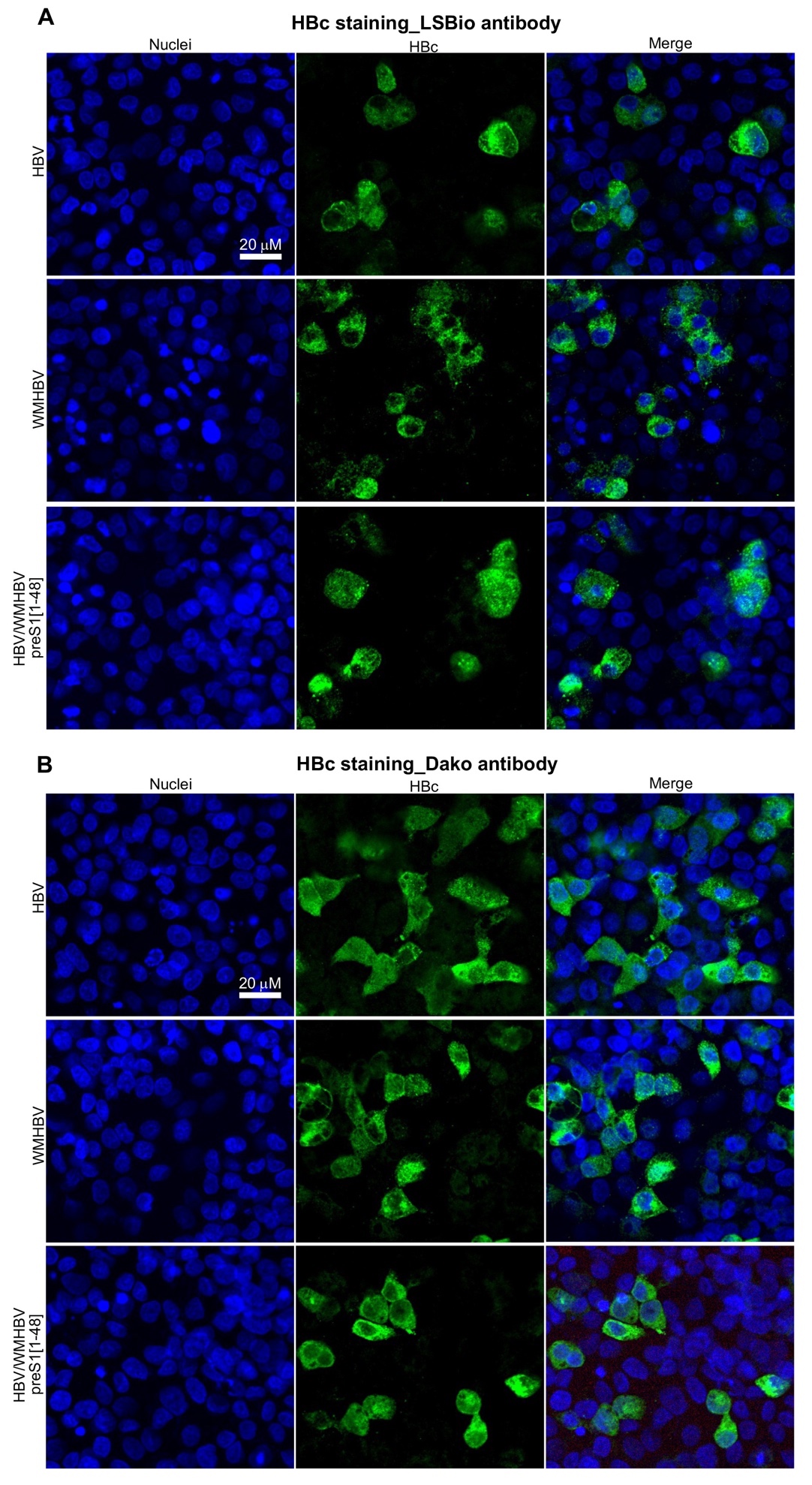


**Supplementary Figure 8.** (**A**) Representative images for immunofluorescence (IF) of HBc after transfection of mock, HBV, WMHBV or HBV/WMHBV plasmid into HepG2 cells. The LSBio HBc antibody was used (1:200 dilution, #LS‑C67477, LSBio, Seattle, WA). The experiments were repeated twice independently with similar results and the representative images were shown. (**B**) Representative images for immunofluorescence (IF) of HBc after transfection of mock, HBV, WMHBV or HBV/WMHBV plasmid into HepG2 cells. The Dako HBc antibody was used (1:500 dilution, #B0586, Dako, Denmark). Images under different fluorescence channels (405 nm and 488 nm) and merged images are shown. The slides from one experiment were viewed under the microscope and 3 images from different fields were taken from which representative images are shown.


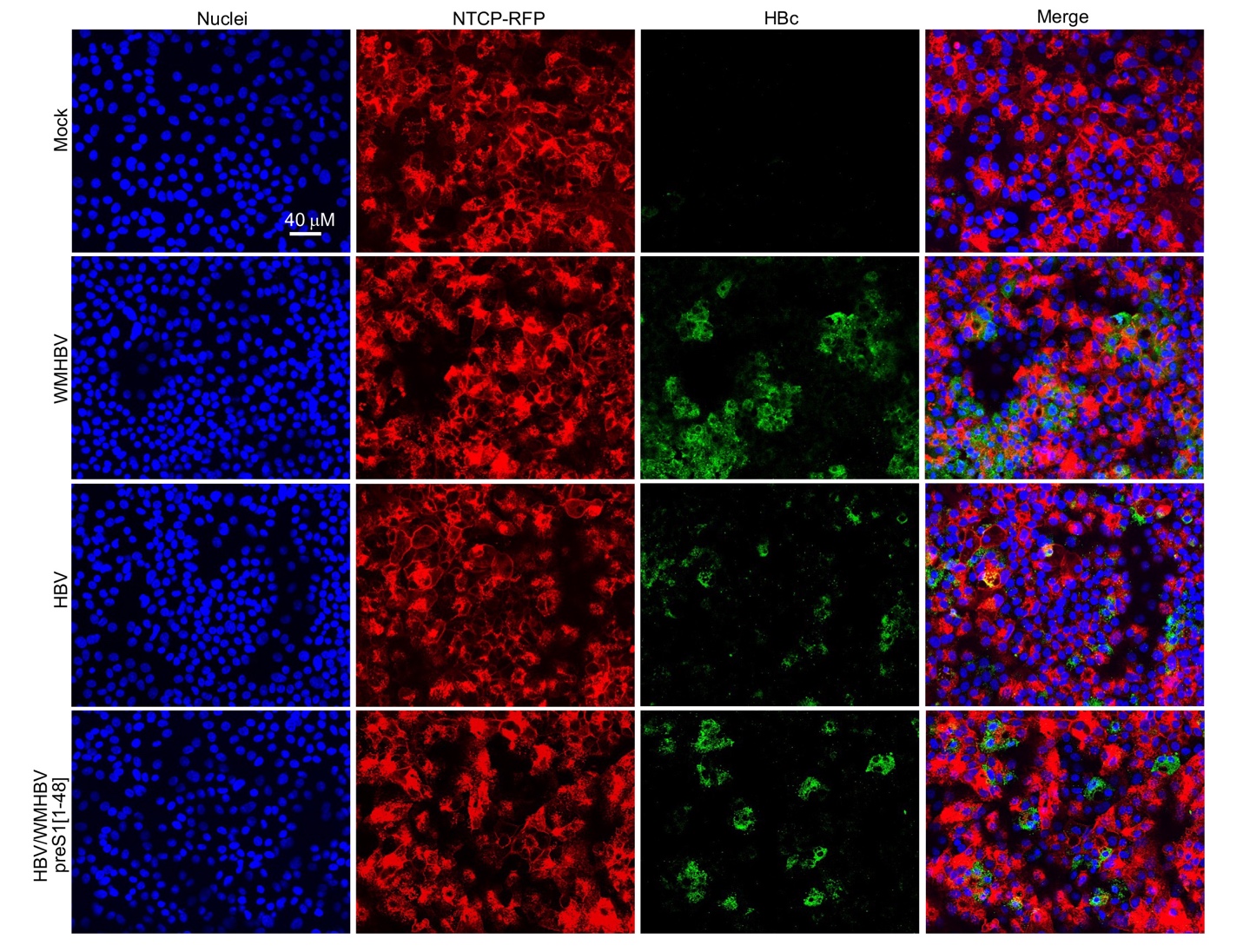


**Supplementary Figure 9.** Representative images for IF of HBc after infection of mock, WMHBV, HBV, HBV/WMHBV preS1[1-48] chimeric viruses on marmoset NTCP-expressing HepG2 cells. Images under different fluorescence channels (405 nm, 488 nm and 561 nm) and merged images are shown. The experiments were repeated twice independently with similar results and representative images are shown.

**Supplementary Figure 10.** (**A**) Three plex combined in suit hybridization (ISH)-immunohistochemistry (IHC) chromogenic assay at the control groups. Representative images of double negative control (naïve HepG2 cells), NTCP positive control (marmoset NTCP expression HepG2 cells with mock infection) and HBV RNA&HBc positive control (HepG2 cells transfected with 1.3x HBV infectious clone). Note all three control samples were placed onto a single slide to evaluate the protocol across all three samples simultaneously ensuring assay specificity. (**B**)Top row, HBV RNA ISH probe was replaced by an internal house-keeping gene (UBC) or a Bacillus subtilis (dapB) gene. Positive signal for UBC confirmed RNA integrity and methodology, while absence of ISH reaction with dapB confirmed assay specificity. All images acquired at 400x total magnification.


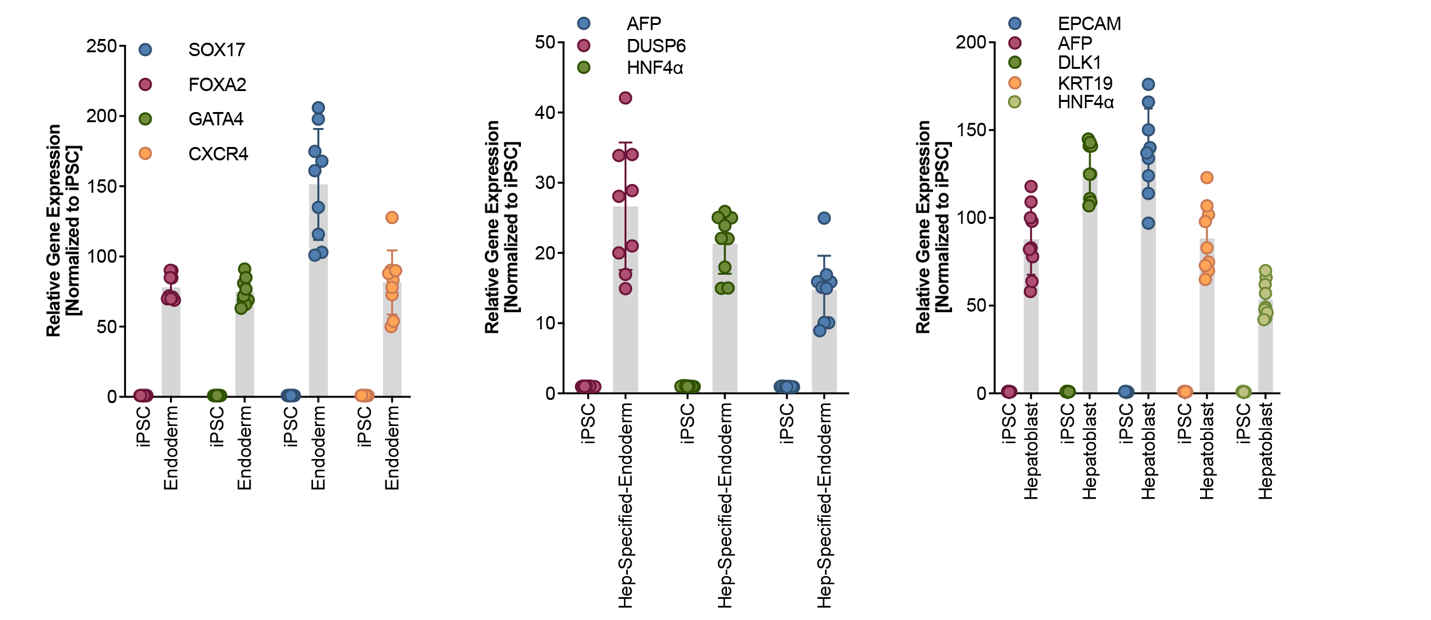


**Supplementary Figure 11.** The stepwise differentiation of the iPSCs characterized by specific genes expression at Endoderm, Hep-Specified-Endoderm and Hepatoblast stages, respectively as previously described^2^. Gray bars depict the mean of 9 biologically independent experiments and error bars represent s.e.m.


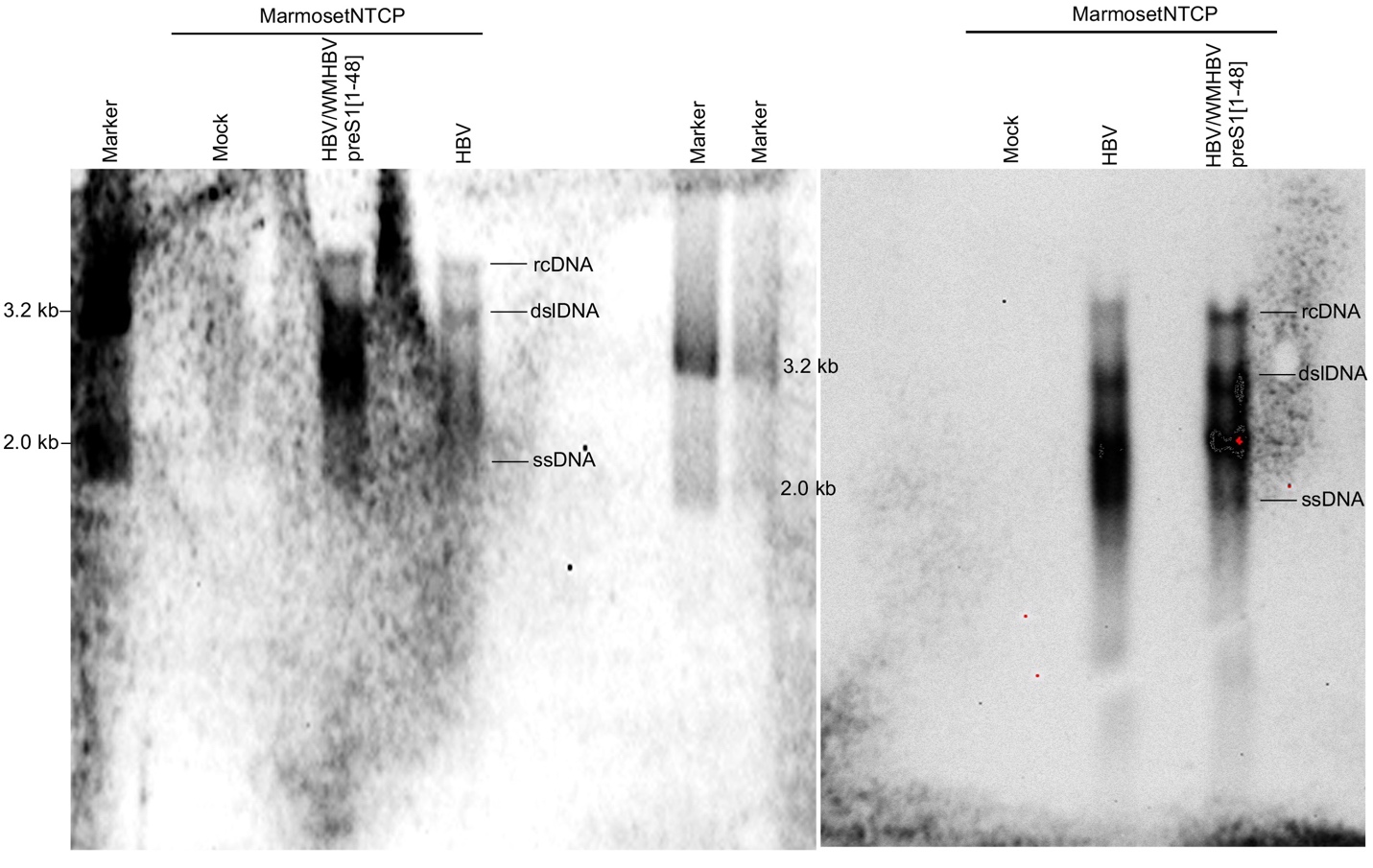


**Supplementary Figure 12.**  Southern bot assay of intracellular HBV DNA replication intermediates after infection. The gel data showed two independent experiments.

**Supplementary References:**

1 Jumper, J. *et al.* Highly accurate protein structure prediction with AlphaFold. *Nature* **596**, 583-589, doi:10.1038/s41586-021-03819-2 (2021).

2 Song, W. *et al.* Engraftment of human induced pluripotent stem cell-derived hepatocytes in immunocompetent mice via 3D co-aggregation and encapsulation. *Scientific reports* **5**, 16884, doi:10.1038/srep16884 (2015).
