# Supplementary material for: Targeted viral adaptation generates a simian-tropic hepatitis B virus that infects marmoset cells
Source: Nat Commun. 2023 Jun 16;14:3582. doi: 10.1038/s41467-023-39148-3 (PMC10276007; doi:10.1038/s41467-023-39148-3)
Supplement: Supplementary file 3 — Reporting Summary [file 41467_2023_39148_MOESM3_ESM.pdf]

Corresponding author(s): Alexander Ploss

Last updated by author(s): May 11, 2023

Reporting Summary

Nature Portfolio wishes to improve the reproducibility of the work that we publish. This form provides structure for consistency and transparency in reporting. For further information on Nature Portfolio policies, see our Editorial Policies and the Editorial Policy Checklist.

Statistics

For all statistical analyses, confirm that the following items are present in the figure legend, table legend, main text, or Methods section.

n/a	Confirmed	
☐	☒	The exact sample size (*n*) for each experimental group/condition, given as a discrete number and unit of measurement
☐	☒	A statement on whether measurements were taken from distinct samples or whether the same sample was measured repeatedly
☐	☒	The statistical test(s) used AND whether they are one- or two-sided<br>*Only common tests should be described solely by name; describe more complex techniques in the Methods section.*
☒	☐	A description of all covariates tested
☒	☐	A description of any assumptions or corrections, such as tests of normality and adjustment for multiple comparisons
☐	☒	A full description of the statistical parameters including central tendency (e.g. means) or other basic estimates (e.g. regression coefficient) AND variation (e.g. standard deviation) or associated estimates of uncertainty (e.g. confidence intervals)
☐	☒	For null hypothesis testing, the test statistic (e.g. *F*, *t*, *r*) with confidence intervals, effect sizes, degrees of freedom and *P* value noted<br>*Give P values as exact values whenever suitable.*
☒	☐	For Bayesian analysis, information on the choice of priors and Markov chain Monte Carlo settings
☒	☐	For hierarchical and complex designs, identification of the appropriate level for tests and full reporting of outcomes
☒	☐	Estimates of effect sizes (e.g. Cohen's *d*, Pearson's *r*), indicating how they were calculated

Our web collection on statistics for biologists contains articles on many of the points above.

Software and code

Policy information about availability of computer code

Data collection	NCBI; PDB; AlphaFold algorithm, Version 2.1.2; BD FACSDiva v9.0;
Data analysis	Prism 9; FlowJo 10; MegAlign Pro, Version: 17.3.1 (11); Protean 3D, Version: 17.3.2 (13), DM Version: 17.3.0 (58); ImageJ, Version: 2.1.0/1.53c; TM-Align, Version 20190822; Biorender (Biorender.com);<br>The script used to generate the AlphaFold prediction has been deposited in Zenodo with the access link https://zenodo.org/record/7799411#.ZC16cHvMJD8.

For manuscripts utilizing custom algorithms or software that are central to the research but not yet described in published literature, software must be made available to editors and reviewers. We strongly encourage code deposition in a community repository (e.g. GitHub). See the Nature Portfolio guidelines for submitting code & software for further information.

Data

Policy information about availability of data

All manuscripts must include a data availability statement. This statement should provide the following information, where applicable:

- Accession codes, unique identifiers, or web links for publicly available datasets
- A description of any restrictions on data availability
- For clinical datasets or third party data, please ensure that the statement adheres to our policy

Data availability: Data are available in the main text or the supplementary materials. Source data are provided with this paper. Human NTCP CDS, NCBI accession

number: JQ814895.1; CynoNTCP CDS, NCBI accession number: AK240620.1; SqMNTCP CDS, NCBI accession number: XM_003924480.3; Marmoset NTCP CDS, NCBI accession number: XM_035260831.1; Prosimian NTCP CDS, NCBI accession number: XM_012656155.1; Human Cryo-EM NTCP structure, PDB:7PQG and 7PQQ. The AlphaFold related structure data and the script used to generate the prediction have been deposited in Zenodo with the access link https://zenodo.org/record/7799411#.ZC16cHvMJD8.

Human research participants

Policy information about studies involving human research participants and Sex and Gender in Research.

Reporting on sex and gender	N/A
Population characteristics	N/A
Recruitment	N/A
Ethics oversight	N/A

Note that full information on the approval of the study protocol must also be provided in the manuscript.

Field-specific reporting

Please select the one below that is the best fit for your research. If you are not sure, read the appropriate sections before making your selection.

☒ Life sciences ☐ Behavioural & social sciences ☐ Ecological, evolutionary & environmental sciences

For a reference copy of the document with all sections, see nature.com/documents/nr-reporting-summary-flat.pdf

Life sciences study design

All studies must disclose on these points even when the disclosure is negative.

Sample size	To do the statistics, at least three biologically independent experiments were performed in this study.
Data exclusions	No data was excluded in the data analysis.
Replication	At least three biologically independent experiments were performed in this study. All attempts at replication were successful regardless of data quality.
Randomization	The samples were allocated into experimental groups randomly.
Blinding	Not blinded. The experimental groups were divided into certain groups by the investigators and the data also were analyzed by the investigators. Since the control groups were always included in the experiments and the experiment results were interpreted by analyzing and comparing the data between control and experimental groups, blinding was not relevant/performed in the study.

Reporting for specific materials, systems and methods

We require information from authors about some types of materials, experimental systems and methods used in many studies. Here, indicate whether each material, system or method listed is relevant to your study. If you are not sure if a list item applies to your research, read the appropriate section before selecting a response.

Materials & experimental systems

n/a	Involved in the study	
☐	☒	Antibodies
☐	☒	Eukaryotic cell lines
☒	☐	Palaeontology and archaeology
☒	☐	Animals and other organisms
☒	☐	Clinical data
☒	☐	Dual use research of concern

Methods

n/a	Involved in the study	
☒	☐	ChIP-seq
☐	☒	Flow cytometry
☒	☐	MRI-based neuroimaging

Antibodies

Antibodies used	anti-HBc (for IF), #LS-C67477, LSBio, Seattle, WA; Alexa Fluor 488 conjugated goat anti-mouse secondary antibody, #A28175, Thermo

Fisher Scientific, Waltham, MA; anti-HBc antibody, #B0586, Dako, Denmark; Alexa Fluor 488 conjugated goat anti-mouse secondary antibody, #A11008, Thermo Fisher Scientific, Waltham, MA; anti-HBc (for IHC), #LS-c312204, LSBio, Seattle, WA; anti-RFP, #R10367, Thermo Fisher Scientific, Waltham, MA; alkaline phosphatase-conjugated DIG antibody, #11093274910, MilliporeSigma, Rockville, MD

Validation	The validation of the antibodies was performed by HBV replication competent plasmid transfection (data provided in the manuscript in Supplementary Fig. 8 and Supplementary Fig. 11.

Eukaryotic cell lines

Policy information about cell lines and Sex and Gender in Research

Cell line source(s)	293 T (American Tissue Culture Collection, ATCC® Number: CRL-3216TM, Manassas, VA;<br>HepG2 cell lines (American Tissue Culture Collection, ATCC® Number: HB-8065™, Manassas, VA);<br>3T3-J2 cells (CCL-92, ATCC, Manassas, VA);
Authentication	They were authenticated by short tandem repeat profiling.
Mycoplasma contamination	Free of mycoplasma contamination.
Commonly misidentified lines (See ICLAC register)	No commonly misidentified cell lines were used in the study.

Flow Cytometry

Plots

Confirm that:

☒ The axis labels state the marker and fluorochrome used (e.g. CD4-FITC).

☒ The axis scales are clearly visible. Include numbers along axes only for bottom left plot of group (a 'group' is an analysis of identical markers).

☒ All plots are contour plots with outliers or pseudocolor plots.

☒ A numerical value for number of cells or percentage (with statistics) is provided.

Methodology

Sample preparation	HepG2 cells or HepG2 cells expressing different NTCPs were seeded in each well of the round-bottom 96-well plates and were cultured in 100 µL pretreatment medium (2% DMSO, 1% Pen/Strep and 1 NEAA in DMEM medium) with 0, 50, 100 and 200 nM peptides, respectively. For competitively inhibition assays, 200 nM peptides and 750 nM Myrcludex B (MyrB, kindly provided by Stephan Urban, University of Heidelberg) were included in the medium. After 1 hour incubation in 37 ºC, unbounded peptides were washed out by FACS buffer (PBS with 1% (vol/vol) FBS) followed by 4% (vol/vol) PFA fixing at 4 ºC for 30 mins. The cells were then washed for another two times and resuspended in 200 µl FACS buffer. NTCP expression and HBV/WMHBV binding were analyzed by flow cytometry to determine tagRFP expression and FITC signal, respectively using a LSRII Multi-Laser Analyzer (BD, Franklin Lakes, NJ) at the Princeton flow cytometry core facility.
Instrument	LSRII Multi-Laser Analyzer (BD, Franklin Lakes, NJ)
Software	BD FACSDiva v9.0; FlowJo v10.0;
Cell population abundance	After gating, about 1000 cells or more targeted cell population was acquired/counted.
Gating strategy	The cells were gated by SSC and FSC first to select the HepG2 or HepG2-NTCP cells. Then a FSC-A and FSC-H gate was used to select the single cells, and the single cells were double purified by SSC-A and SSC-H. Then a SSC-A and mCherry-A gate was used to select NTCP-RFP positive cells and then SSC-A and FITC-A gate was used to select the target population. Corresponding controls were used to divide the gate boundary.

☒ Tick this box to confirm that a figure exemplifying the gating strategy is provided in the Supplementary Information.
